# A Hierarchical Approach to Activity Recognition and Fall Detection Using Wavelets and Adaptive Pooling

**DOI:** 10.3390/s21196653

**Published:** 2021-10-07

**Authors:** Abbas Shah Syed, Daniel Sierra-Sosa, Anup Kumar, Adel Elmaghraby

**Affiliations:** 1 Department of Computer Science and Engineering, University of Louisville, Louisville, KY 40208, USA; anup.kumar@louisville.edu (A.K.); adel.elmaghraby@louisville.edu (A.E.); 2 Department of Computer Science and Information Technology, Hood College, Frederick, MD 21701, USA; sierra-sosa@hood.edu

**Keywords:** smart health, Internet of Things (IoT), artificial intelligence, activity recognition, cyber physical systems, fall detection, direction and severity

## Abstract

Human activity recognition has been a key study topic in the development of cyber physical systems and assisted living applications. In particular, inertial sensor based systems have become increasingly popular because they do not restrict users’ movement and are also relatively simple to implement compared to other approaches. In this paper, we present a hierarchical classification framework based on wavelets and adaptive pooling for activity recognition and fall detection predicting fall direction and severity. To accomplish this, windowed segments were extracted from each recording of inertial measurements from the SisFall dataset. A combination of wavelet based feature extraction and adaptive pooling was used before a classification framework was applied to determine the output class. Furthermore, tests were performed to determine the best observation window size and the sensor modality to use. Based on the experiments the best window size was found to be 3 s and the best sensor modality was found to be a combination of accelerometer and gyroscope measurements. These were used to perform activity recognition and fall detection with a resulting weighted F1 score of 94.67%. This framework is novel in terms of the approach to the human activity recognition and fall detection problem as it provides a scheme that is computationally less intensive while providing promising results and therefore can contribute to edge deployment of such systems.

## 1. Introduction

Fall detection is an important task in the care of elderly who are more likely to suffer from a fall compared to young people and sometimes may even die from it [1]. According to the World Health Organization [2], falls are the second leading cause of unintentional injury worldwide and within the US; a fall is experienced every second by people aged above 65 years old [3]. Moreover, the likelihood of experiencing more falls increases after the undergoing the first fall event [4]. The ageing population (by 2050, the population of people aged 60 and above will increase to 2.1 billion according to the United Nations [5]) of the world presents in itself a challenge for providing healthcare services effectively, not only in terms of the capacity and capability to deliver it to the population but also in terms of high costs related to fall based injuries (running to the tune of $50 Billion annually [6]).

People can suffer falls due to a number of ailments, such as visual impairments, cardiovascular diseases, cognitive impairments and and illnesses such as parkinsons, arthritis, epilepsy, etc. In such situations, a fall detection system (FDS) can be an important tool in the provision of healthcare to people and have been used for assisting in health care provision as well [7,8]. The aim of a FDS is to monitor the movement of a person and determine when a fall has taken place with the aim to alert healthcare personnel or other caregivers. These systems can be vital in some situations, for, e.g., authors in [9] note that fall detection systems are necessary for old people with cognitive impairments who may not be able to get up after a fall for long durations of time which may result in pressure sores and other complications.

Development of FDS takes two routes based on the environment they are deployed in, these two types are context aware systems (CAS) and non-context aware systems (non-CAS). CAS systems detect falls by sensing the environment as a whole, of which the user is a part of. Such systems include Ambient sensor based FDS and Vision based FDS. Ambient sensor based FDS make use of various ambient sensors such as human presence infrared sensors [10] and other sensors for the environment etc as their sensing modality. On the other hand Vision based FDS use devices such as video cameras [11] and Kinect [12] as their sensing equipment. CAS FDS have the limitation that they are only usable in a small setting, such as a room or a nursing home. This is due to the fact that they are expensive to deploy and maintain and also due to a fixed deployment, they could possibly restrict freedom of movement for the user. Moreover, due to the inherent nature of the sensing scheme, there may be various issues that need to be overcome when using them for fall detection purposes like occlusion for vision based FDS and spurious sensor triggers for Ambient FDS.

The other type of fall detection systems are Wearable FDS which fall in the category of non-CAS FDS. Wearable FDS typically include the use of sensors such as movement sensors (accelerometers, gyroscopes), pressure sensors [13] or sensors measuring health related signals (ECG [14], EEG [15], EMG [16]) attached to a body. Data from these sensors can then be used to determine if a fall has occurred or not. Many times, Wearable FDS make use of multiple units attached to a body in order to better capture the movement patterns of a user. In contrast to CAS FDS, Wearable FDS do not restrict movement of the subjects and therefore are more user friendly. Moreover, most wearable FDS sensors, especially movement sensors, are inexpensive and are present in many electronics such as smartphones and smart watches. Such systems are easy to deploy, thus making wearable FDS development popular for fall detection purposes. Wearable FDS consisting of accelerometers and/or gyroscopes can be deployed using a persons phone or as independent units attached to the body. These sensors continuously monitor the persons movement patterns and process the data gathered by the sensors to determine whether a fall has taken place or not. Data from the sensors is first processed before it can be used, processing might involve filtering of the signals, extracting sliding windows of observation and possible feature extraction. Once the signals have been processed, they are passed on to a decision making system or algorithm. In this regard, machine and deep learning systems have garnered the most interest of researchers as such algorithms are able to learn the nonlinear relationships between the various activities or falls to determine the desired outcome.

In this work, we provide a framework for a fall and activity recognition system. The framework aims to differentiate between various activities of daily living as well as various types of falls with regard to fall direction and severity aware. To do this, we make use of data from the SisFall dataset [17] and after suitable pre-processing and feature extraction, make use of machine learning algorithms to differentiate between different activities of daily living (ADL) and falls.

This paper is organized as follows, Section 2 provides a discussion of the related literature, Section 3 discusses the data used in the work, Section 4 elucidates on the experimental setup with results presented in Section 5 and a discussion provided in Section 6. Lastly, Section 7 concludes the work.

## 2. Literature Review

Post fall intelligence is an important research area in the field of fall detection as it can be useful in determining various post fall injuries [18] and serve as an intelligence parameter [19] for doctors. Koo et al. [20] present experiments for post fall detection from a combination of self collected data and the SisFall dataset. They conduct tests using sliding windows as well as discrete windows from these signals and compute statistical features from them. After feature extraction, two different classifiers, the Artificial Neural Network (ANN) and Support Vector Machine (SVM) are tested with the computed features as well as raw sensor values. They find that both ANN and SVM are suitable for use in post fall detection scenarios. Another approach looking at the different phases of a fall has been presented in [21] where Hsieh et al. use accelerometer sensor data to differentiate between five phases of a fall, pre-fall, free-fall, impact, resting and recovery and the initial and end static phases. To do this, they compute various time domain and statistical features and test five classifiers, SVM, K-Nearest Neighbors (KNN), Naive Bayes (NB), Decision Trees (DT) and Adaptive Boosting (AdaBoost). For their experimental setup, the best results were achieved using the KNN classifier. A different take on post fall intelligence is the determination of direction in falls. Direction aware fall detection has been performed by Hossain et al. in [22,23] where they include ADLs along with direction sensitive fall detection using an accelerometer. In their work, they use various statistical features from the data collected by them along with an SVM classifier to different between five different classes, ADL, Forward Fall, Backward Fall, Right Fall and Left Fall. More work on direction aware fall detection has been performed by Lee et al. [24] who use data from an accelerometer with thresholding, and by Lee. J.K. [25] who makes use of kalman filters to determine the tilt angles from accelerometer and gyroscope data along with an SVM and by Tolkiehn et al. [18] using an accelerometer and barometer along with thresholding. Direction determination within the fall detection has also been a researched problem in some methodologies proposed in the domain of pre-impact fall detection where falls are detected inorder to trigger a protection device. Ahn et al. [26] develop a pre-impact fall detection system using data from the SisFall dataset. They use acceleration, angular velocity, vertical angle and a ‘traingular feature’ (formulated by them) along with thresholding to determine directions in the pre-impact part of falls. While direction aware fall detection is an important determination in terms of post fall intelligence, fall detection with severity is necessary since it could help provide indications to falls with immediate recovery or otherwise, as falls without immediate recovery would be more detrimental to health than a fall with immediate recovery as has been suggested by Palmerini et al. [27].

In [28], Hussain et al. propose a fall detection system that can first determine falls and then the type of fall using data from the SisFall dataset. They accomplish this in a hierarchical setup where their system first considers fall detection as a binary problem, whether a fall has taken place or not, and if a fall has been detected, it classifies between the various falls in the dataset. Their system is designed to work with 10 s non-overlapping windows of accelerometer and gyroscope signals. Data from each record are first low pass filtered before two different types of feature sets, consisting of various time domain and statistical features, are computed on the data. This is then followed by the machine learning stage where three different classifiers are tested, KNN, SVM and Random Forests (RFC). In the fall detection stage, statistical features are computed from ADL and fall signals and sent to the three classifiers for the preliminary binary classification. After a fall has been determined to have happened, numerous other statistical and time domain features are then computed on the data before being sent to the next stage to determine the type of fall activity taking place. In their experiments, the authors find that KNN is most effective in differentiating between falls and ADLs where as RFC performs the best when the different fall activities need to be determined. They achieve an F1 score of 99.75% and 79.95%, respectively, for their setup. This work highlights the usefulness of a hierarchical approach towards non-binary fall detection. An interesting approach towards fall detection while considering fall direction and severity has been proposed by Gibson et al. [29] where the authors use multiple classifiers to vote for any of the considered classes. They use accelerometers to gather data for ADLs and various fall types and compute wavelet coefficients from the data using a debauchies level-3 wavelet. First, eight intermediate classes are formed from the four output classess and five different classifiers (ANN, KNN, Radial Basis Function Network (RBF), Probabilistic Principal Component Analysis (PPCA) and Linear Discriminant Analysis (LDA)) are trained which vote for that particular event to have taken place. For each event, fusion through majority voting is used as an indicator for a given event to have happened. This information, for each event, is then passed on to a second stage that consists of a comparator machine which evaluates these event indicator results based on a set of rules and with help from a supervisory KNN multiclass classifier. The authors in this work consider fall detection with direction and severity with good results, however, they perform their experiments on a self collected dataset with the different falls being performed from a standing position. This does not necessarily represent a real world situation where a person might be performing different activities before undergoing the fall.

Various feature extraction schemes have been used in the area of activity recognition and fall detection, including, time/frequency domain and statistical features [30], different wavelet transforms [31,32] and even raw sensor signal values being used with deep learning networks [33]. In [34], Abdu-Aguye and Gomaa present a method combining wavelet transform and adaptive pooling to perform activity recognition. Spatial Pyramid Pooling [35] is an adaptive pooling method which was developed to address the issue of fluctuating input sizes in CNNs for image-based applications, and it entails converting varying-size convolutional feature maps into fixed-length summarizations. These summarizations, having uniform length can then be passed on to the fully connected parts of the CNN where a fixed length input is necessary. Given a pooling size *pxp*, adaptive pooling works by dividing the input in to *pxp* pieces while computing the size of each piece automatically and performing any necessary padding. Once these pieces are created, a pooling operation is typically performed (max pooling or average pooling for e.g.,) on each of these pieces to summarize the input into an output of fixed size *pxp*. This results in a fixed output length for any size of the input. Abdu-Aguye and Gomaa [34] find that the combination of adaptive pooling with wavelets as input features for machine learning algorithms produces results comparable to using a CNN fed with raw sensor signal data.

It can be observed that while fall detection has been looked at in a more in-depth manner then the case of a binary detection scheme (fall vs. no fall), very little work has been carried out in the detection of falls with direction and severity. Keeping this in mind, in our work in [36], fall detection with direction and severity was performed using a combination of time and frequency domain features and an SVM classifier using data from the SisFall dataset. However, in that work, fall detection was considered as an isolated task. In this work, we consider the problem of fall detection with direction and severity in the light of formal human activity recognition, in that, we aim to differentiate between different activities of daily living and fall types as a holistic problem. Furtheremore, from a fall only perspective, we improve on the average F1 score compared to our previous approach. Lastly, the hierarchical methodology proposed here is tested on a public dataset. The framework presented here is novel in terms of its approach to the problem, by identifying and utilizing a feature extraction scheme that is computationally simple and adding to it a classification structure that simplifies the problem at hand, it provides promising results for a problem that has not been addressed in great detail in previous research work.

## 3. Data

The SisFall dataset was released by the Universidad de Antiquia to support research in the fall detection domain [17]. Their dataset is an extensive repository consisting of recordings of falls and activity of daily living (ADL) performed by 38 participants. In total, 19 ADLs and 14 falls were performed with 5 trials for each Fall and ADL except the activites of walking and jogging. In total, the total number of ADL recordings in the dataset are 2707 and Falls are 1798. For all recordings in the dataset, measurements were collected by a sensing unit placed at the waist of the participants consisting of two accelerometers (ADXL345 from Analog Devices and MMA8451Q from Freescale Semiconductor (Freescale Semiconductor, Austin, TX, USA)) and one gyroscope (ITG3200 from Texas Instruments (Texas Instruments, Dallas, TX, USA)) at a sampling rate of 200 Hz.

In this work, we make use of the SisFall dataset to perform fall detection with direction and severity and activity of daily living detection since it has been the dataset of choice in multiple works addressing the fall detection domain [37,38,39] as it includes recordings of volunteers from various ages (ages from 19 to 75 years), has diversity in the gender make up of the participants (19 males and 19 females from a total of 38 volunteers) and is one of the biggest datasets available in terms of the type of falls and activities being recorded. Since both accelerometers are placed at the same position and therefore measure the same movements, data from only one of the accelerometers along with the gyroscope are considered in this work. In addition, since we aim to perform activity recognition and fall detection with direction and severity, the labeling of the original dataset has been modified. This labeling has been shown in Table 1 and Table 2. As can be observed from Table 1, the activities Walking (W), Jogging (J), Sitting (S) and Standing (SB) have been considered for this work which are typical activities in ADL detection problems. Each of these labels includes data from multiple original activities, for, e.g., activities with original labels of walking upstairs and downstairs, walking slowly and walking quickly have been considered as walking in this work. A similar scheme has been used for the other three activity labels as well. Some of the activities such as being on one’s back change to lateral position, wait a moment, and change to one’s back (D14), getting in and out of the car (D17), stumble while walking (D18), and gently jumping without falling while trying to reach a high object (D19) have not been considered. The reason for this is that they have very few samples to be considered as standalone activities (only one type of sub-activity and also because most of these are not considered in typical ADL detection scenarios).

The labeling used for the falls present in the SisFall dataset is presented in Table 2. All the falls in the dataset have been labeled in to two categories, either soft/hard or in to three categories in terms of direction, forward, backward and lateral. It should be mentioned that for two falls, F06 and F07, the falls were labeled using video recordings provided as part of the SisFall dataset. In addition to direction, falls were also labeled separately for their severity. To do this, all falls which included softening the impact using some support were labeled as Soft Falls where as those without were labeled as Hard Falls, a similar approach was used by Gibson et al. [29]. The final labeling for the falls consists of six types when direction and severity are combined, these are Forward Soft Falls (FSF), Forward Hard Falls (FHF), Backward Soft Falls (BSF), Backward Hard Falls (BHF), Lateral Soft Falls (LSF) and Lateral Hard Falls (LHF).

## 4. Methodology

**Figure 1:**
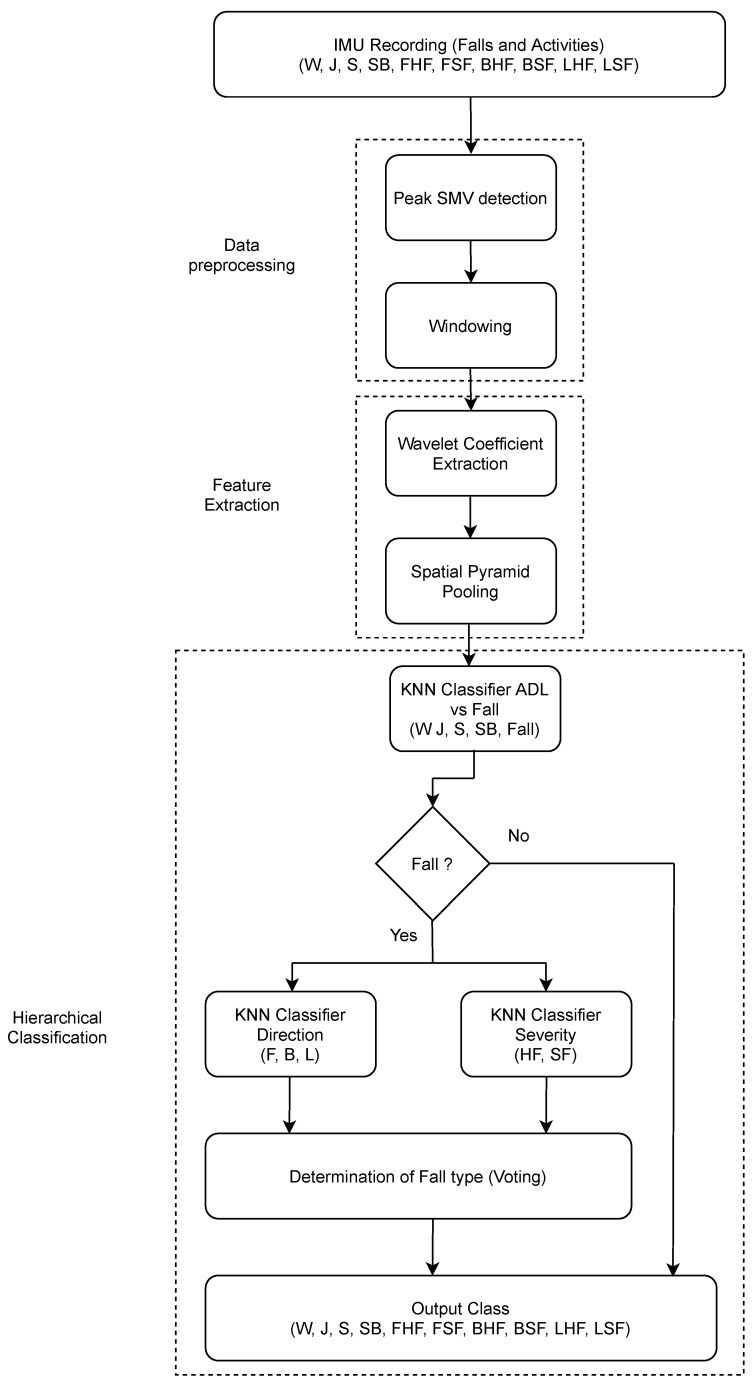
Hierarchical classification scheme for ADL and Fall detection.

The methodology in this work follows the common scheme for a machine learning based solution to activity recognition and fall detection. The first stage consists of data preprocessing, followed by feature extraction and then evaluation or classification. Figure 1 shows the methodology for this work with individual parts being elaborated upon in the proceeding subsections. All preprocessing, feature extraction and classification was performed in Python. The implementation of the machine learning algorithms used was from the Scikit-Learn (https://scikit-learn.org/stable/ accessed on 3 October 2021) libary.

### 4.1. Data Preprocessing

Data preprocessing involves the conversion of the input signals in to a form that is more suitable for use in the later feature extraction stage. Recordings in the SisFall dataset vary in length between 12 and 100 s. In order to perform feature extraction in a uniform manner, it is required that the considered signal be of the same duration, to do this, first we determine the value of the Signal Magnitude Vector (SMV) [33] for all samples in a given activity/fall recording. The SMV can be computed as,

$$SMV_{j}=\sqrt{{\left|A_{x_{j}}\right|}^{2}+{\left|A_{y_{j}}\right|}^{2}+{\left|A_{z_{j}}\right|}^{2}}$$

where $SMV_{j}$ stands for the SMV value for a given sample j in a activity/fall trial. Once the SMV values have been determined for all the samples in a recording, the peak value of the SMV is used as a midpoint to extract a window of duration n seconds around it. Windowed segments are extracted in this manner from all considered activities in the SisFall dataset except the activities of D01, D02, D03 and D04 which consist of a single trial per subject of duration 100 s. In such cases, continuous windowed segments of duration n seconds are extracted from the recordings. It is also pertinent to mention here that since both accelerometers are placed at the same position, we only consider one of the accelerometers along with the gyroscope readings present in the recorded trials. To determine the value of n as well as the appropriate sensor modality to use for the final system, experiments were performed on the developed framework and the results have been discussed in Section 5.

### 4.2. Feature Extraction

**Figure 2:**
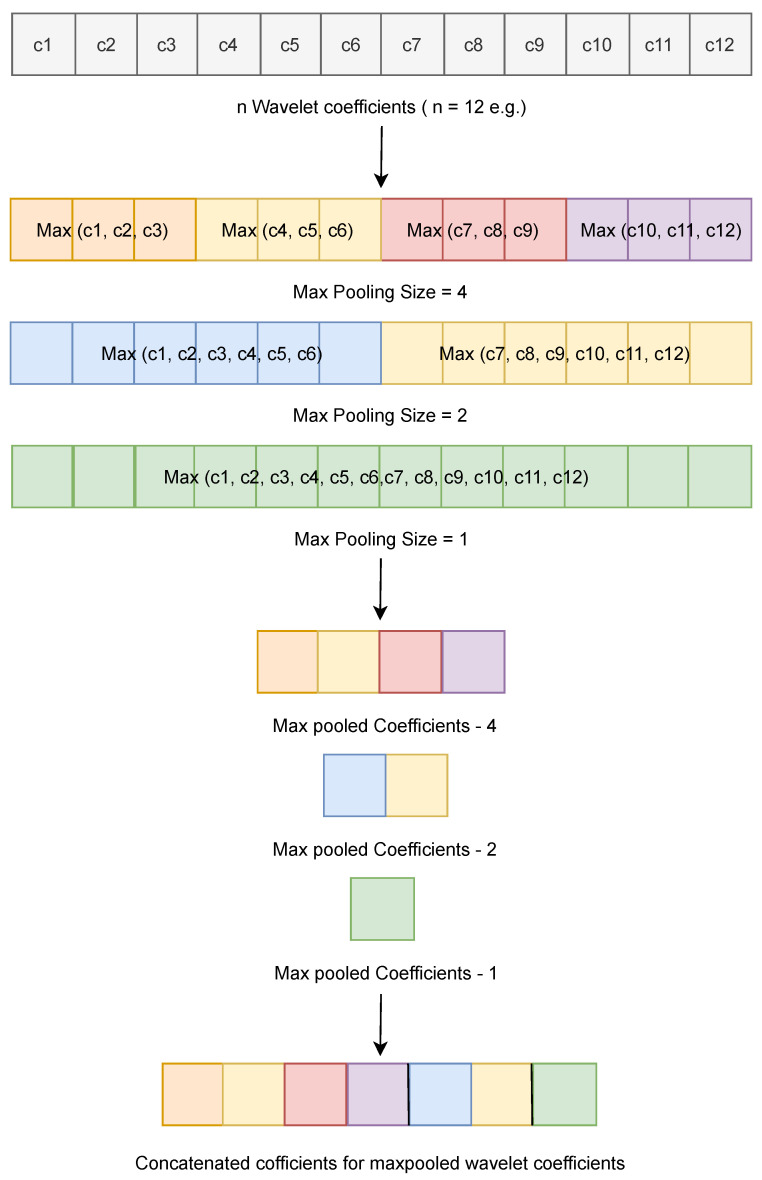
Example: 4-2-1 1-D Spatial Pyramid Pooling.

Feature extraction involves conversion of input in to a form that can effectively be used for discriminating between the different classes at the output. In this work, we utilize haar wavelets along with 4-2-1 1D Spatial Pyramid pooling to extract features from the windowed segments of activity and fall data. First, for each segment, wavelet coefficients are extracted using a haar wavelet. Tests were performed with level values of 2, 3, 4, 5 and 7 and it was determined that level-4 produced the best results. Once detail and approximation coefficients were extracted from the windowed segments, for the set of coeficients, 4-2-1 Spatial Pyramid pooling is performed as illustrated in Figure 2. Each coefficient set was divided in to four and two parts and then max pooling was used to determine the maximum value in these divided parts and the coefficient set as a whole. These maximum values were then concatenated together to form the seven valued output from that coefficient set. Furthermore, the results for each coefficient set within each axis were also concatenated to form the feature vector for a sensor axis measurement. This operation was performed for each axis of accelerometer and groscope sensor data with the final feature vector of 210 values consisting of the concatenations of the individual vectors for each axis. It is hypothesized that this way local as well as global information at each level of the wavelet coefficients can be captured.

### 4.3. Classification

A hierarchical classification approach is employed to discriminate between the various activities and falls considered from the SisFall dataset. Hierarchical classification involves the division of a complex taxonomic classification problem in to a set of subsets that are potentially easier to differentiate as the task becomes more localized. Hierarchical classifiers have been used in multiple different applications [40] where they have been found to improve upon the performance of many flat classification schemes. The classification framework used in this work combines hierarchical classification with a vote based system. The classification problem is divided into three parts, each with its own classifier to indicate to the subclass of the output. The classifier in part one consists of differentiating whether a given recording is a fall or one of the four considered ADLs. In order to train this stage, the activities of Standing, Walking, Sitting and Jogging along with all falls combined in to one class are passed to the classifier. This dilutes the original ten-class problems in to a five-class sub problem. The output of this stage is the determination of whether a given recording is either one of the four ADLs (Standing, Walking, Sitting or Jogging) or a fall. If a recording has been detected to be a fall, it is sent to the second and third stages.

The second and third stages work in parallel on samples detected as falls from the first stage in the form of a voting machine. These two stages vote individually on the direction and severity of the detected fall samples. In order to train them, fall samples were relabeled to represent direction and severity only and are fed to the classifiers. For the direction, the classification problem is formulated as a three-class problem of determining fall directions as being Forward, Backward or Lateral. For the severity classifier, the classification problem is formulated as a two-class problem of a fall being either Soft or Hard. After a signal has passed through all necessary stages, the outputs of the individual stages are combined to indicate to the activity or type of fall being fed at the input.

**Figure 3:**
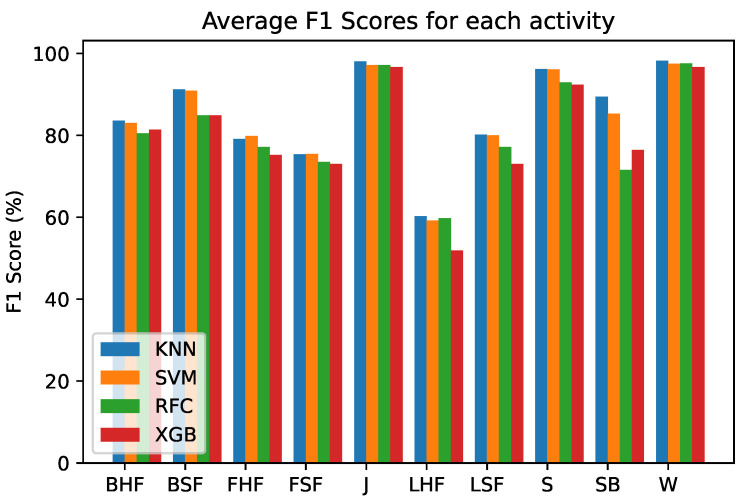
Average F1 Scores for each activity for the four classifiers.

Four classifiers were tested for each part of the hierarchical scheme, the classifiers considered were K-Nearest Neighbors (KNN), Support Vector Machines (SVM), Random Forests (SVM) and eXtreme Gradient Boosting (XGB). Parameter tuning was performed using gradient search for each classifier over a range of values for each parameter. Experiments were performed for the considered window durations for each activity and the classifier which provided the best performance overall was chosen. The mean F1 scores for each output class for each classifier are shown in Figure 3. It can be observed that in general KNN and SVM perform better compared to the ensemble models, the RFC and XGB. However, since the KNN slightly outperforms the SVM in eight of the ten considered classes, we choose KNN as the classifier for this framework.

## 5. Results

The data after the feature extraction stage were split in to a train/test partition based on a 75/25 ratio. As mentioned earlier, the classifiers were trained from a parameter grid to determine best tuning parameters for maximizing the weighted F1 score while using five-fold cross validation. We used the weighted F1 score as our training metric due to the imbalance in the samples of the different classes in the data. Moreover, for evaluation purposes, we report on the individual F1 scores for each output class and provide discussions as necessary. For our best case scenario, we also report on the sensitivity, precision as well as the specificity. The sensitivity/recall or the true positive rate of the proposed system guages the systems capability to identify the correct class, precision or positive predictive value indicates to the correctness of the detected values and specificity or the true negative rate gives an assessment of the system to not miss-classify the class as any of the other classes (these metrics have been computed on a one vs. all basis). Furthermore, in order to determine the best values for the observation window and the most appropriate sensor modality to use, two experiments were conducted with multiple values/combinations for these two parameters.

An important consideration in working with activity recognition systems is to determine the appropriate observation window size for the analysis of sensor signals to accomplish the ADL recognition/fall detection task. The size of the observation window is important as a smaller observation window increases the response time of the activity recognition/fall detection system and it can also impact the time taken in the computation of features. In order to find the best observation window size, we perform experiments using five values, 2, 3, 4, 5 and 6 s. The classification results in terms of the F1 score are presented in Table 3. For each case, samples of duration equal to half of the observation window were extracted around the peak value of the SMV. From the table, it can be observed that an observation window of size 3 s produces the best results for six out of the ten output classes. It only produces poorer results for the classes BHF, BSF and S, SB where window sizes of 2 s, 6 s and 4 s, respectively, perform better than the 3 s windowing case. Upon further investigation of this phenomenon using the result of other classifiers, it was observed that the activities of (BHF and BHF) were best recognized by all the classifiers with a window size of 2 s (for the case of KNN, there is a small difference between the 2 s and 6 s case), for the other two activities of S and SB too the F1 score was obtained for the 4 s duration (for the activity S, the difference in performance over windows larger than 4 s is very small). This could be attributed to the feature aggregation process in the max pooling operation in the different spatial segments.

The second experiment in designing the proposed system is the determination of the best sensor modality to use. Using a single sensor would result in less data, faster processing and reduced hardware costs compared to the multisensor approach combining accelerometer and gyroscope. To do this, the classification framework was tested with 3 s windowed segments of the combined acceleromter and gyroscope data as well as data of the accelerometer and gyroscope sensors individually. The results of this experiment are presented in Table 4. It can be observed that using a combination of both accelerometer and gyroscope data together produces the best results for eight of the ten output classes. An accelerometer-only system produces better results for the detection of activity SB and the fall FHF. The outcome of this experiment agrees with previous work for fall detection by Waheed et al. [37] on the SisFall dataset.

Table 5 reports on the best results obtained for the proposed classification framework. These results were achieved by using windowed segments of 3 s and combined data from the accelerometer and the gyroscope with a weighted F1 score of 94.67% on the test set.

## 6. Discussion

From Table 5, the best recognized ADLs are W and J whereas the best recognized fall is BSF. The worst performing class in ADLs is SB whereas the worst performing fall is LHF. Upon further inspection of the cause of the bad performance with LHF, looking at the confusion matrix, it was observed that LHF falls were most commonly confused with FSF which resulted in a reduction of the classification performance for this class. On the other hand, in the case of FSF (the second worse performing class), looking at the confusion matrix, it was observed that FSF was confused with LSF and FHF. Furthermore, the specificity values indicate that there has been very little mis-identification for each of the classes. When talking about the activity S, it was observed that samples from this activity were confused with the activity W which resulted in the sub par performance of the classifier for its recognition.

To investigate the effectiveness of the proposed scheme, Table 6 provides a comparison of the proposed method to the one presented in [28]. This is the most similar work to the problem being addressed herein in that it presents a hierarchical classification scheme for different types of falls. In order to incorporate ADL classification in their scheme, a separate ADL classification stage was added which works in parallel to the already present fall classifier stage. Furthermore, for this experiment, the data was filtered as in [28] and windowed with a duration of 3 s before being passed on as input to the two schemes. It can be observed from Table 6 that the proposed framework provides better recognition for all of the considered activities of daily living as well as falls. The average F1 scores for the method of [28] is 87.46% where as for the proposed scheme it is 90.76%. This demonstrates the effective performance of the proposed scheme in terms of being useful for the problem of combined ADL recognition and fall detection with severity and direction determination.

There are several directions for future work. Given that it was found that a combination of accelerometer and gyroscope sensor measurements produced the best results in the proposed setup, one of the approaches that could be useful to improve upon the current scheme is sensor fusion. Here, data from multiple sensors, possibly non-movement sensors such as EEG as noted by Wang et al. [41] or ECG measurements, can be fused together to improve performance of fall detection systems. This would help in developing systems that provide additional information about a patients health apart from performing fall detection only. Another addition in this regard would also be the addition of sensor data from other positions on the body that can capture the different movement patterns in a different manner and to combine data from various positions.

Another area of work would be to use deep learning methods for this application. Deep Learning (DL) models such as Convolutional Neural Networks can be used to extract ‘interesting’ features from sensor data that conventional feature extraction methods are unable to do, thereby having the potential of providing better performance for such tasks in the classification stage. Recurrent Neural Network could also be used to learn from the movement patterns generated from the inertial sensors. A limitation to the use of DL methods for this application currently is the lack of data; however, such limitations could be mitigated by the use of data augmentation schemes, transfer learning and the use of cross dataset experimentation. These methodologies could result in improved performance for the task discussed.

## 7. Conclusions

Human activity recognition has been an important research area of cyber physical system development and assisted living applications. In this regard, the usage of inertial sensor data has been very popular as they do not restrict a users movement and are also easy to deploy compared to other methods.

Utilizing inertial sensor data, in this paper, a hierarchical classification framework using wavelets and adaptive pooling has been presented for the purpose of activity recognition and fall detection considering fall direction and severity. To achieve this, inertial sensor recordings (accelerometer and gyroscope) from the SisFall dataset were utilized and windowed segments were extracted from each recording. Following this, a level-4 haar wavelet was used to extract wavelet coefficients from these windowed segments and then 4-2-1 1-D Spatial Pyramid pooling was used to summarize the output of the wavelet feature coefficients at each approximation and detail level before the max pooled coefficients were concatenated to form the final feature vector. A hierarchical classification scheme was then used consisting of three classification stages, one for determining individual ADLs vs. a generic fall and the second and third for fall direction and severity, respectively, with both voting together to determine the severity and direction of a fall. Towards this end, experiments were conducted to determine the most appropriate size of the observation window as well as sensing modality used. It was found that for the proposed setup, a window duration of 3 s produced the best results while using data from both the accelerometer and gyroscope.

## Figures and Tables

**Table 1:** Labeling used for Activities in the SisFall dataset.

SisFall	Assigned	Assigned
Activity Code	Activity Name	Activity Label
D01	Walking	W
D02	Walking	W
D03	Jogging	J
D04	Jogging	J
D05	Walking	W
D06	Walking	W
D07	Sit	S
D08	Sit	S
D09	Sit	S
D10	Sit	S
D11	Sit	S
D12	Sit	S
D13	Sit	S
D14	-	-
D15	Standing	SB
D16	Standing	SB
D17	-	-
D18	-	-
D19	-	-

**Table 2:** Labeling used for Falls in the SisFall dataset.

SisFall	Assigned Fall Name			Assigned
Fall Code	Direction Only	Severity Only	Direction + Severity	Fall Label
F01	Forward Fall	Hard Fall	Forward Hard Fall	FHF
F02	Backward Fall	Hard Fall	Backward Hard Fall	BHF
F03	Lateral Fall	Hard Fall	Lateral Hard Fall	LHF
F04	Forward Fall	Hard Fall	Forward Hard Fall	FHF
F05	Forward Fall	Hard Fall	Forward Hard Fall	FHF
F06	Forward Fall	Soft Fall	Forward Soft Fall	FSF
F07	Lateral Fall	Soft Fall	Lateral Soft Fall	LSF
F08	Forward Fall	Soft Fall	Forward Soft Fall	FSF
F09	Lateral Fall	Soft Fall	Lateral Soft Fall	LSF
F10	Forward Fall	Soft Fall	Forward Soft Fall	FSF
F11	Backward Fall	Soft Fall	Backward Soft Fall	BSF
F12	Lateral Fall	Soft Fall	Lateral Soft Fall	LSF
F13	Forward Fall	Soft Fall	Forward Soft Fall	FSF
F14	Backward Fall	Soft Fall	Backward Soft Fall	BSF
F15	Lateral Fall	Soft Fall	Lateral Soft Fall	LSF

**Table 3:** Performance for different observation window sizes.

Activity	Observation Window Size (F1 Score [%])				
	2 s	3 s	4 s	5 s	6 s
BHF	86.79	83.02	79.25	83.64	85.19
BSF	92.17	90.76	89.08	90.76	**93.22**
FHF	78.53	80.47	78.32	79.21	78.83
FSF	73.39	77.18	72.5	76.83	76.79
J	97.53	98.27	98.08	98	98.16
LHF	52.83	67.8	62.75	59.26	58.62
LSF	79.69	82.73	77.57	81.46	79.41
S	95.27	96.2	97.6	95.84	95.93
SB	87.29	85.71	91.98	90.61	91.71
W	98.08	98.46	98.12	98.35	98.16

**Table 4:** Performance for different sensing modalities.

Activity	Sensing Modality (F1 Score [%])		
	Accelerometer + Gyroscope	Accelerometer	Gyroscope
BHF	83.02	67.92	82.14
BSF	90.76	85.48	78.18
FHF	80.47	83.33	71.17
FSF	77.18	73.21	63.96
J	98.27	97.79	95.59
LHF	67.8	54.55	55.56
LSF	82.73	76.34	73.21
S	96.2	95.61	91.17
SB	85.71	86.21	76.09
W	98.46	98.24	96.3

**Table 5:** Best Results (Obs. Window: 3 s, Sensing Modality: Acc. + Gyro.).

Activity	Precision (%)	Sensitivity (Recall) (%)	Specificity (%)	F1-Score (%)
BHF	95.65	73.33	99.96	83.02
BSF	91.53	90	99.8	90.76
FHF	86.08	75.56	99.57	80.47
FSF	76.86	77.5	98.88	77.18
J	97.87	98.68	99.36	98.27
LHF	68.97	66.67	99.65	67.8
LSF	79.85	85.83	98.96	82.73
S	95	97.44	99.31	96.2
SB	93.75	78.95	99.8	85.71
W	97.95	98.97	98.36	98.46

**Table 6:** Comparison of proposed scheme to the work in [28].

Activity	F1 Score (%)	
	Method of [28]	Proposed Scheme
BHF	87.72	**93.1**
BSF	94.02	**97.44**
FHF	83.06	**87.21**
FSF	81.15	**82.2**
J	96.5	**98.27**
LHF	62.22	**73.33**
LSF	85.83	**87.3**
S	96.83	**97.13**
SB	89.13	**92.63**
W	98.14	**99.05**

## Data Availability

The dataset used in this work is publicly available at: http://sistemic.udea.edu.co/en/research/projects/english-falls/.
